# Biopsy proteome-based classification of T cell-mediated kidney allograft rejection

**DOI:** 10.1186/s12967-025-07116-8

**Published:** 2025-10-21

**Authors:** Daria Kania, Daniel Fochtman, Łukasz Skoczylas, Marta Gawin, Łukasz Marczak, Agata Kurczyk, Katarzyna Fidyk, Agnieszka Perkowska-Ptasińska, Ewa Chmielik, Alicja Dębska-Ślizień, Justyna Gołębiewska, Anna Wojakowska, Monika Pietrowska

**Affiliations:** 1 Center for Translational Research and Molecular Biology of Cancer, Maria Skłodowska-Curie National Research Institute of Oncology Gliwice Branch, Wybrzeże Armii Krajowej 15, 44-102 Gliwice, Poland; 2 Institute of Bioorganic Chemistry, Polish Academy of Sciences, Noskowskiego 12/14, 61-704 Poznań, Poland; 3 Department of Biostatistics and Bioinformatics, Gliwice Branch, Maria Skłodowska-Curie National Research Institute of Oncology, Wybrzeże Armii Krajowej 15, 44-102 Gliwice, Poland; 4 Tumor Pathology Department, Gliwice Branch, Maria Skłodowska-Curie National Research Institute of Oncology, Wybrzeże Armii Krajowej 15, 44-102 Gliwice, Poland; 5 Department of Pathology, Medical University of Warsaw, Ul. Pawińskiego 3B, 02-097 Warsaw, Poland; 6 Department of Nephrology, Transplantology and Internal Medicine, Medical University of Gdańsk, Smoluchowskiego 17, 80-952 Gdansk, Poland

**Keywords:** Kidney transplant rejection, Borderline rejection, T-cell mediated rejection, Mass spectrometry-based proteomics, Immunohistochemical staining

## Abstract

**Background:**

T cell-mediated rejection (TCMR) remains a challenge in kidney transplantation. Based on a histopathological biopsy examination, patients can be classified into groups such as no rejection (NR), borderline rejection (BR; Banff category 3), and acute rejection (AR; Banff category 4). Yet, this classification is not sufficient, since for the borderline cases a number of patients may require a clinical intervention. Thus, a robust classification by biopsy proteome profiling may provide a solution.

**Methods:**

In this work, kidney tissue from patients classified into NR, BR, and AR were subjected to MS-based proteomic profiling. Subsequently, a panel of four proteins (GNB4, PDK1, AGXT, CD73) was selected for validation by immunohistochemistry (IHC). This retrospective study was approved by the Bioethics Committee of the Medical University of Gdańsk, no. NKBBN/201/2021.

**Results:**

Proteomic analysis identified 2547 proteins whose abundance profiles demonstrated strong concordance between the BR and AR groups. In a quantitative comparison between the BR and AR groups, GNB4 and AGXT emerged as significantly differentiating. Moreover, AGXT was indicated as a potential biomarker following ROC analysis. PDK1 and CD73 were found to best classify the samples in a binary analysis. IHC confirmed only upregulation of GNB4 in immune cells and PDK1 in macrophages, with no significant changes in the tubular epithelium.

**Conclusions:**

Thus, GNB4 and PDK1 in immune cells and macrophages have been identified as a potential target for further extensive studies. If their relevance were to be confirmed in a larger patient cohort, their IHC analysis could serve as an extension of established histopathological classification in the context of kidney transplant rejection.

**Supplementary Information:**

The online version contains supplementary material available at 10.1186/s12967-025-07116-8.

## Introduction

Enhanced understanding of the immune system role in allograft rejection along with advancements in immunosuppressive therapy have enabled more effective organ transplantation; however, T cell-mediated rejection (TCMR) remains a common complication [1, 2]. The kidney allograft function decline, which may occur shortly after transplantation or even years later, usually manifests clinically with decreased urine output and proteinuria, with more severe cases including transplant tenderness. Even though TCMR responds well to immunosuppressive therapy, rejection still proceeds in around 10–12% of patients [3]. Kidney allograft biopsy, evaluated according to the Banff classification, remains the gold standard for diagnosing rejection [4, 5]. This grading system includes the classification of cases into “*category 4: TCMR”,* with its subcategories including “*acute TCMR”*, and also defines certain cases as “*category 3: borderline (suspicious) for acute TCMR*” [5].

Borderline rejection is a difficult diagnostic category. The diagnosis is made upon the assessment of the areal extent of interstitial inflammation and tubulitis using arbitrary thresholds. The thresholds are broad (especially depending on the edition of the Banff criteria) expanding from minor or absent interstitial infiltration (Banff i1and i0) to 10–25% cortical inflammation with foci of various degree tubulitis (Banff t1, t2, and t3). What is more, both interstitial inflammation and tubulitis are nonspecific lesions associated with various conditions in both native and transplanted kidneys. Both the lack of pathognomonic qualitative criteria and broad quantitative criteria result in borderline rejection being potentially a heterogenous diagnostic grouping [6–8].

There have been conflicting reports on the clinical significance of borderline cases. They are associated with increased numbers of subsequent clinical interventions; however in many cases, the pathological changes may also resolve spontaneously [6, 9–12]. Although borderline rejection may indicate inadequate control of the alloimmune response, it remains unclear whether all patients require anti-rejection treatment and which interventions are most effective in preventing unfavorable outcomes. Some cases of borderline rejection may actually represent acute rejection with discrepancies arising from sampling errors and inter-observer variability in assigning Banff scores [11, 12]. For this reason, there is an ongoing search for tools to improve the diagnostic accuracy of an inherently imperfect histopathological assessment [13].

Molecular alterations precede visible structural changes and, if quantitatively assessed, they offer an immense diagnostic potential. Proteomic analysis allows for the detection and quantification of thousands of proteins, facilitating the identification of changes associated with allograft rejection and providing insights into the underlying biological processes. This approach has been successfully applied before, enabling the discrimination between acute and no rejection patients with high reliability (AUC = 0.87) based on a panel of three serum proteins proposed by Wang et al. [13]. Epidermal growth factor analysis in the urine of patients was proposed as a potential biomarker of antibody-mediated rejection (AUC = 0.78) in a study reported by Heidari et al. [14]. Yet, in an expanded multicenter study enrolling 635 patients, discrimination for acute TCMR based on urine analysis yielded subpar results [15]. It is possible that, thus far, noninvasive analyses have given unsatisfactory or unrepeatable results because proteins that would reflect allograft rejection status are low-abundant components of urine or blood (as compared to other highly abundant proteins, *e.g.*, serum albumin). Thus, a robust method of TCMR classification is still needed, which could be achieved via mass spectrometry (MS) based analysis of biopsy specimens. This study aimed to identify possible proteomic signatures of both borderline for acute TCMR (BR) and acute TCMR (AR), (collectively labeled ‘R’—rejection), using kidney allograft biopsy specimens (FFPE material) and to validate selected biomarker candidates based on immunohistochemical methods (used as a standard procedure during a histopathological examination).

## Materials and methods

### Patient samples

Retrospective FFPE kidney allograft tissue cores were obtained at the Medical University of Gdańsk Transplant Center. For this retrospective proteomic study, samples were obtained from patients who underwent a for-cause biopsy – performed due to worsening kidney function or proteinuria, or erythrocyturia—between 2014 and 2022. Based on histopathological assessment of the collected material, patient samples were later classified into three groups: no rejection (NR; *n* = 10), borderline rejection (BR; *n* = 12), and acute rejection (AR; *n* = 10). All biopsies were classified according to the current Banff criteria at the Department of Pathology, Medical University of Warsaw, Poland. For the immunohistochemistry staining, due to the limited amount of material, a separate set of samples of deceased kidney donors’ preimplantation biopsies was obtained. Thus, for the immunohistochemistry analysis, the grouping was as follows: donor kidney (DK; *n* = 8), borderline rejection (BR_IHC; *n* = 8), and acute rejection (AR_IHC; *n* = 9). Fully anonymized clinical data for these sample groups are available as supplementary tables (see Additional File 1 and Additional File 2). No statistically significant correlation was found between the available clinical data and the biopsy-related group assignments.

### Sample preparation for proteomics analysis

To the FFPE tissues, 0.5 mL of heptane was added, and the samples were incubated for 30 min at room temperature and spun down at 16,000 g for 2 min. Heptane was discarded, and this de-waxing step was repeated three times. Finally, 50µL of methanol was added, samples were centrifuged at 16,000 g for 2 min, and the supernatant was discarded. The remaining pellet was air-dried for 20 min. Proteins were extracted from the FFPE tissues according to a modified procedure used by Geoui et al. [16] and using the Qproteome FFPE Tissue Kit (Qiagen). The pellet was reconstituted with 100µL of extraction buffer, then incubated at 100 °C for 20 min and at 80 °C for 2 h with 750 rpm mixing. After cooling to 4 °C, samples were centrifuged at 20,000 g for 15 min at 4 °C, and the extraction buffer was topped up to 125µL. Next, 400µL of methanol, 100µL of chloroform, and 300µL of water were added, and each time the samples were mixed and spun down. The supernatant was discarded, and 300µL of methanol, then 1 mL of acetone, was added, with mixing, spinning down, and supernatant removal. The air-dried protein pellet was resuspended in 50µL of 0.1% RapiGest (Waters Corporation) in 50 mM ammonium bicarbonate (AmBIC) and heated to 100 °C for 20 min. Following centrifugation at 20,000 g for 15 min, the supernatant protein concentration was determined as described by Wiśniewski and Gaugaz [17]. Next, 50µL of 50 mM dithiothreitol in 25 mM AmBIC was added, and the samples were incubated at 37 °C for 1 h with 950 rpm mixing. Alkylation was performed using 50µL of 100 mM iodoacetamide in the dark for 1 h. Samples were digested with a trypsin-to-protein ratio of 1:50 for 18 h at 37 °C in 150µL of AmBIC. After digestion, 45µL of 5% trifluoroacetic acid was added. Samples were incubated with 45µL of 5% trifluoroacetic acid at 37 °C with agitation at 950 rpm for 60 min and centrifuged at 18,000 g for 30 min. After desalting, peptides were vacuum-dried and re-dissolved in 20µL of water. Quantification of peptides was performed as per Wiśniewski and Gaugaz [17]. Finally, samples were acidified to 0.1% (v/v) TFA for analysis.

### Mass spectrometry-based proteomics

Peptides were separated using an Acclaim PepMap RSLC nanoViper column (75 µm × 25 cm, 2 µm particle size) with a 190 min gradient of 4 – 60% acetonitrile in 0.1% formic acid at 300 nl/min using a Dionex UltiMate 3000 RSLC nanoLC system. Mass spectrometry analysis was performed using Exploris 480 Orbitrap (ThermoFisher) with top-10 data-dependent acquisition in positive ionization at MS resolution of 70,000 and MS/MS of 17,500. Peptides with ≥ 2 charges in the 300–2000 m/z range were fragmented at normalized collision energy of 28% using higher energy collisional dissociation (HCD). Raw data was processed using Protein Discoverer v.2.2 (Thermo Fisher Scientific) based on the Uniprot database with 10 ppm MS and 0.08 Da MS/MS accuracy. Methionine oxidation, carbamidomethylation, and two missed digestion sites were allowed. Proteins were identified if at least two peptides per protein at the false-discovery rate (FDR) ≤ 0.01 were found. Total ion current was used as a normalization factor.

### Statistical analysis

Missing values were imputed with random numbers from a lognormal distribution with parameters estimated by the maximum likelihood method while accounting for the missing tail (truncation between 0 and the lowest measured value for a particular protein). The Kruskal–Wallis test with post hoc Conover comparisons was used for quantitative analyses [18] with the Jonckheere − Terpstra test for trend analysis [19]. Effect size was calculated as eta-squared with standardized Conover test, yielding Pallant’s “r” [20] with Cohen’s interpretation, *i.e.*, small (|*r*|> 0.1), medium (|*r*|> 0.3), and a large effect (|*r*|> 0.5) [21]. Fisher’s exact test was used for binary analysis with Cramér’s V as an effect size measure and the Cochran-Armitage test for trend [22]. The Benjamini–Hochberg multiple testing correction was applied, and the statistical significance threshold was set to 0.05. Identified proteins were considered as differentially expressed /accumulated when the p-value after FDR correction was lower than 0.05. Analyses were conducted using R v.4.3.1 and MatLab v.2023a. ROC analysis was performed using MetaboAnalyst 6.0 (www.metaboanalyst.ca) with balanced sub-sampling, Monte Carlo cross-validation, and feature ranking by Linear Support Vector Machine and a built-in algorithm.

### Immunohistochemistry staining

The FFPE tissues, provided in 1.5 mL Eppendorf tubes, were initially deparaffinized and subsequently embedded individually in separate paraffin blocks. Sectioning of these tissue blocks was performed using the Microm HM 355S microtome (Thermo Fisher Scientific). Slides of 4 µm thickness were deparaffinized and hydrated, then epitope retrieval was performed using the DAKO PT Link instrument (Agilent Technologies) at preheat temperature 85–97 °C for 10 min, epitope retrieval at 97 °C for 20 min and a cooldown to 75 °C for 20 min. Subsequently, EnVision FLEX Target Retrieval Solution diluted 1:50 (Agilent Technologies) was used as follows: HIGH pH for GNB4, PDK1, CD73, and LOW pH for AGXT. The slides were treated with Flex Peroxidase Blocking solution for 10 min, primary antibody for 20 min, Flex HRP for 15 min, Flex DAB with Substrate-Chromogen for 10 min, and Flex hematoxylin for approximately 45 s. Antibodies were diluted 1:300 for AGXT (#22,394–1-AP, Proteintech), CD73 (#PA5-29,750, Invitrogen), GNB4 (#PA5-113,327, Invitrogen) and 1:100 for PDK1 (#MA5-32,702, Invitrogen) using the FLEX Antibody Diluent. EnVision FLEX Wash buffer diluted 20 × was applied before the addition of each reagent for 10 min. One slide for each patient was stained with hematoxylin and eosin (H&E). Digitization was performed using a Pannoramic 250 slide scanner with the Pannoramic Viewer software v.1.15 (3DHISTECH).

## Results

### Proteomic profiling of kidney tissues

**Fig. 1:**
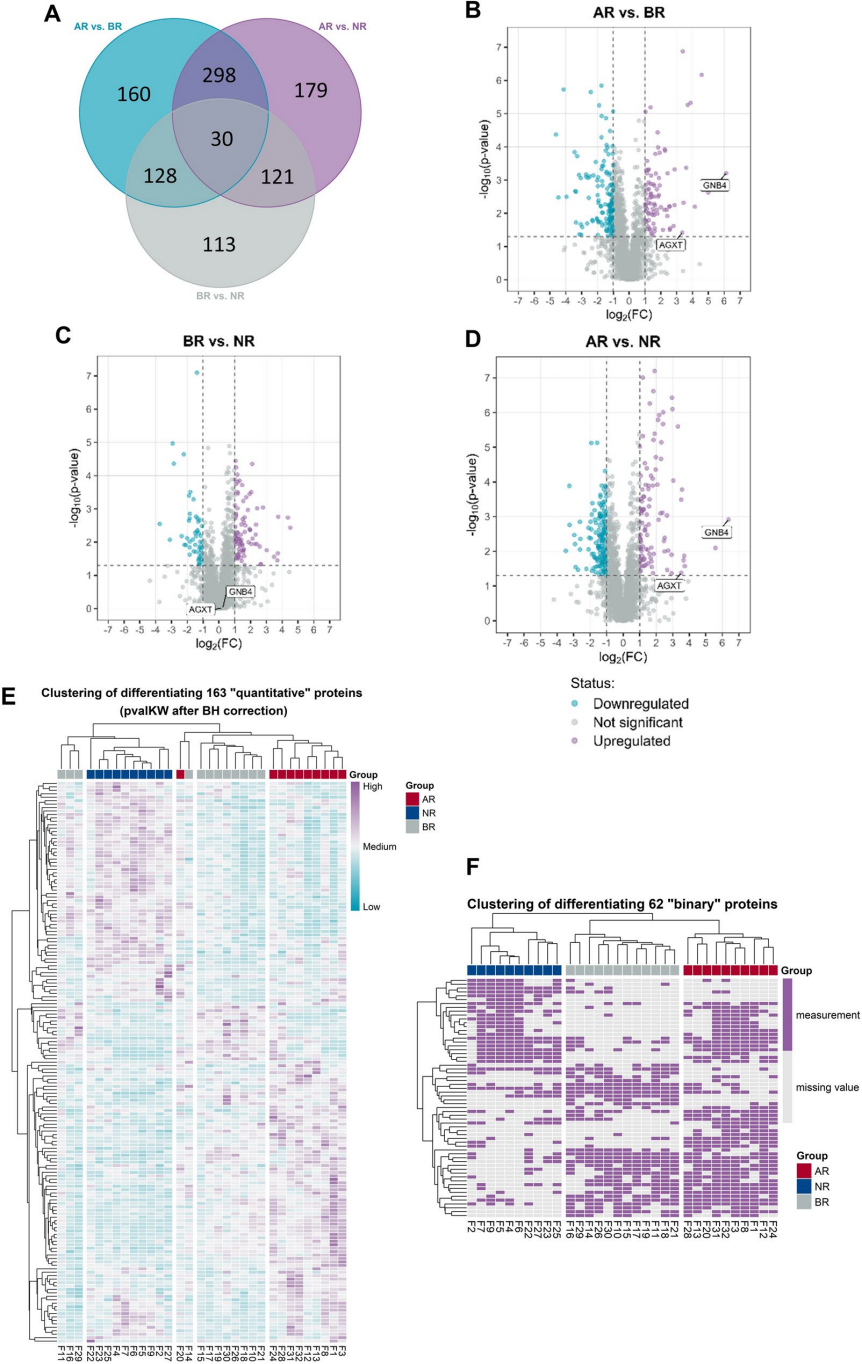
Proteomic profiling of kidney tissues from patients with kidney transplant showing no signs of rejection (NR), with borderline (BR) or acute rejection (AR). **A** Venn diagram indicating the number of differentiating proteins between experimental groups. **B, C, D** Volcano plots denoting the statistical significance and fold-change for all identified proteins in all possible group comparisons. Upregulated proteins are marked in purple, whereas downregulated proteins are marked in blue. Proteins chosen for further study due to their quantitative differentiation (i.e., AGXT, GNB4) are marked separately. **E**, **F** Heatmaps for proteins differentiating the experimental groups in a quantitative and binary manner

Proteomic profiling of kidney tissues in a retrospective analysis of biopsies from patients without (NR) and with borderline (BR) or acute transplant rejection (AR) allowed the identification of 2547 proteins in total. Out of these, quantitative analysis was possible for 2273 proteins, whereas 274 proteins were included in a binary type of analysis (*i.e.*, present/absent). The complete list of identified and quantified proteins is presented as a supplementary table (see Additional File 3). 1029 differentially expressed proteins (DEPs) were identified (FDR < 0.05): 392 between BR and NR groups, 628 between AR and NR, and 616 between AR and BR (Fig. 1A, 1, 1, 1). To find proteins upregulated in both borderline and acute rejection (as compared to the no-rejection group), further analysis was focused on analyzing common proteins that differentiated AR or BR from NR. This comparison resulted in 225 proteins commonly differentiating AR and BR from NR groups (163 in a quantitative manner and 62 in a binary fashion; Fig. 1E and 1, respectively). Noteworthy, hierarchical clustering of the proteomic data revealed a very similar profile of samples in the AR and BR groups.

**Fig. 2:**
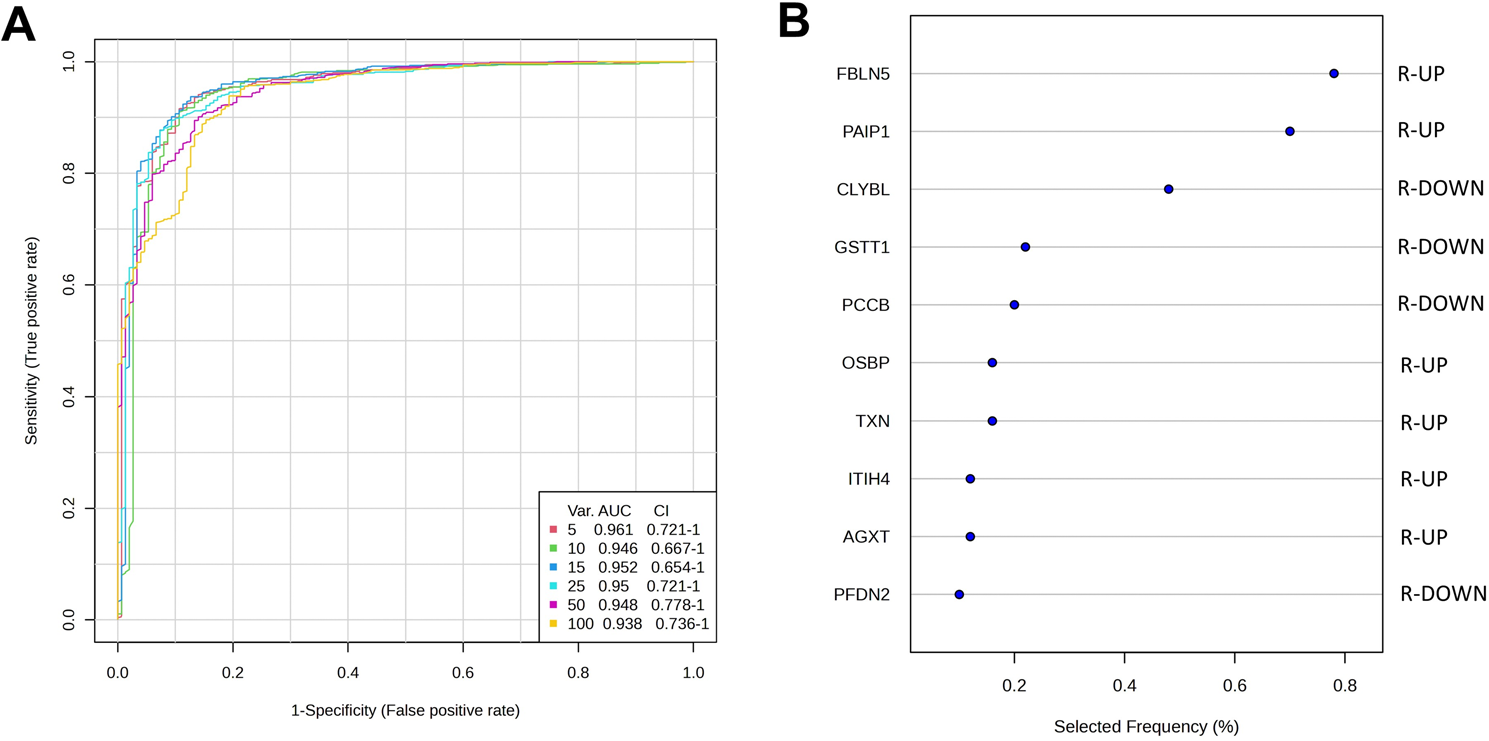
Prediction of potential proteomic biomarker by ROC analysis. **A** Multivariate ROC curves for six models differing in the number of proteins (from 5 to 100) as their variables in comparisons of NR vs R (rejection). **B** Frequency of the proteins present in the model based on the top-ten features (6 proteins upregulated in R and 4 proteins upregulated in NR)

A hypothetical panel of potential proteomic biomarkers of TCMR was tested using a multivariate approach (Fig. 2A). Due to the similarity of proteomic profiles in the AR and BR groups, a model comparing NR vs both AR and BR (collectively labeled ‘R’—rejection) was constructed. Here, a set comprising ten proteins was indicated as the one with the best predictive accuracy (88.2%) with an AUC of 0.946. Notably, AGXT, which was chosen for further validation (see below), was among proteins having a high predictive value based on the ROC analysis (Fig. 2B). Additional panels for paired comparisons are available as a supplementary figure (see Additional File 4).

**Fig. 3:**
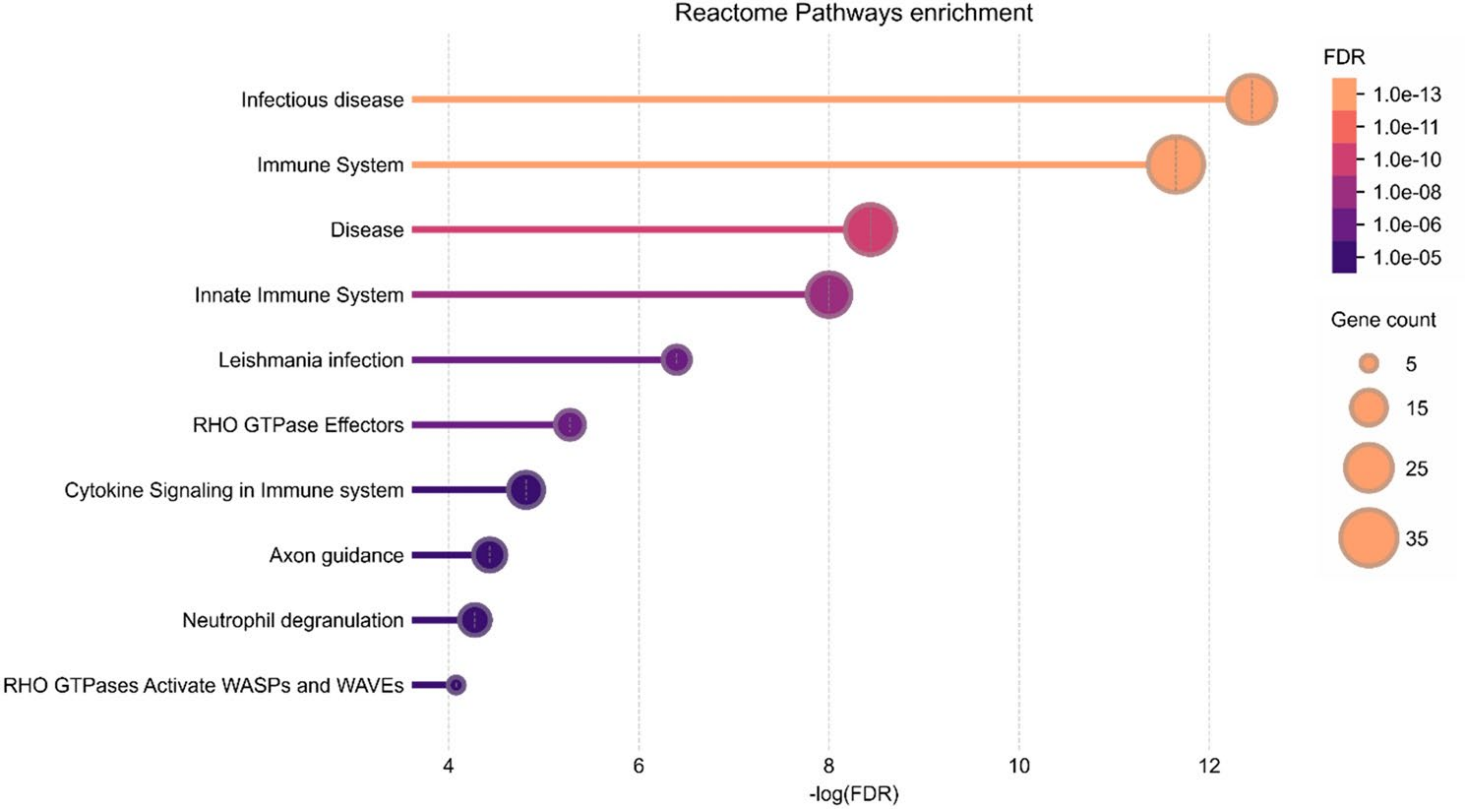
In silico functional analysis of upregulated proteins common to AR and BR groups. Reactome Pathways enrichment plot showing ten most significantly upregulated pathways associated with the differentiating proteins

To further narrow down the proteins associated with the kidney transplant rejection, we focused on the subset of 80 proteins upregulated in both AR and BR (as compared to NR) that showed a large effect size (|*r*|> 0.5) of differences supplementary figure (see Additional File 5). These proteins were characterized functionally in silico. Among processes linked to the subset of AR/BR-upregulated proteins, the most significantly overrepresented were: *response to stimulus*, *positive regulation of biological process*, *cellular component organization* and *immune system process* (see Additional File 6). Further, the Reactome Pathways enrichment analysis revealed *infectious disease*, *neutrophil degranulation*, *immune system* and *innate immune system* as the most significantly overrepresented process. Moreover, this subset of proteins included species linked to RHO-GTPase pathway effectors and cytokine signaling (Fig. 3).

**Fig. 4:**
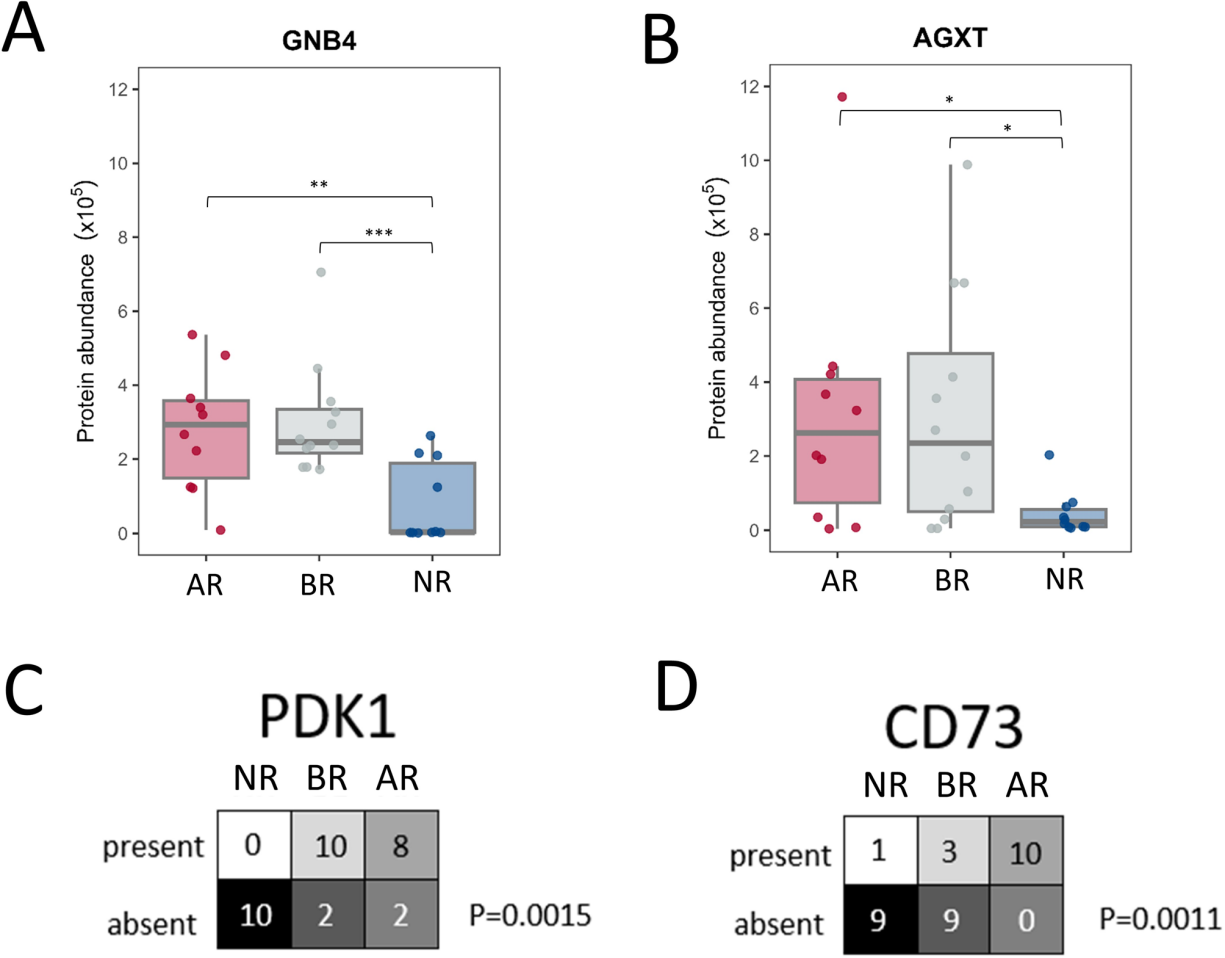
Proteins selected for further study with immunohistochemical staining. **A, B** Bar plots showing quantitative protein abundance differences between the experimental groups for GNB4 and AGXT. **C, D** Binary protein abundance differences (present/absent) for PDK1 and CD73. Statistical significance is denoted as: * *p* < 0.05, ** *p* < 0.01, *** *p* < 0.001

Among the 80 proteins significantly upregulated in the AR/BR groups, guanine nucleotide-binding protein subunit beta-4 (GNB4) exhibited the highest relative abundance, with approximately 80-fold upregulation compared to the NR group (Fig. 4A). Next, alanine-glyoxylate aminotransferase protein (AGXT) was about tenfold upregulated compared to the NR group. ROC analysis further identified it as a key protein with strong predictive value. Although GNB4 did not appear in the list of most frequently chosen proteins for the prediction of transplant status, its high upregulation makes it an ideal candidate for a biomarker, which warrants further validation using IHC. Moreover, two proteins had the most predominant presence in the AR and BR groups as compared to the NR group based on the binary analysis: PDH kinase 1 (PDK1) and 5’-nucleotidase (CD73) (Fig. 4C, 4). Hence, these four proteins were selected for further validation study using immunohistochemical methods.

### Immunohistochemical validation of selected proteins characteristic of transplant rejection

**Fig. 5:**
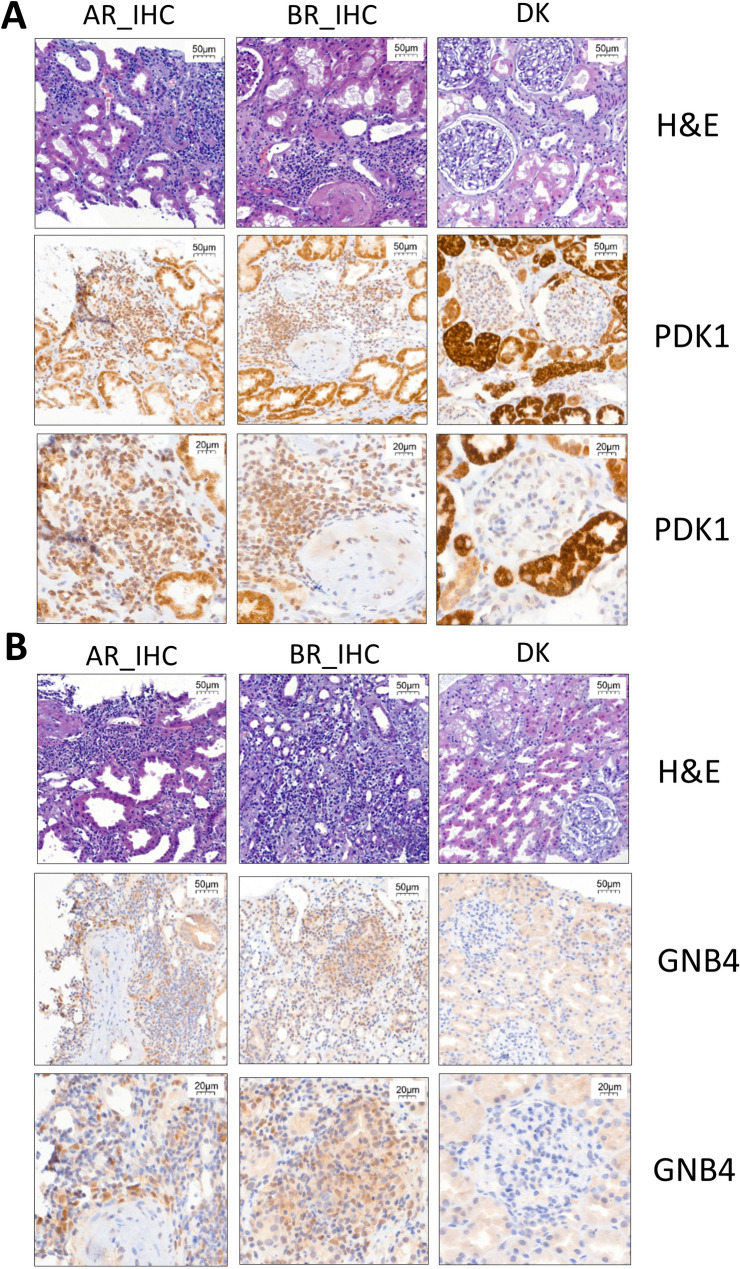
Representative images for H&E and immunohistochemical staining. Tissue sections were stained for PDK1 (**A**) and GNB4 (**B**)

Four proteins (GNB4, AGXT, PDK1, and CD73) putatively discriminating patients with a transplant rejection, as identified by proteomic profiling, were validated using the immunohistochemical approach in a set of kidney biopsies from healthy kidney donors (DK) and patients with borderline (AR_IHC) and acute (AR_IHC) transplant rejection. Localization of these proteins in tissue sections was determined, *i.e.*, staining intensity and area in either tubular epithelium, immune cells, and/or macrophages were assessed (Fig. 5). Out of four proteins selected for immunohistochemical analysis, neither AGXT nor CD73 yielded statistically significant differences between the experimental groups. Hence, the presentation of results was focused on GNB4 and PDK1.

**Fig. 6:**
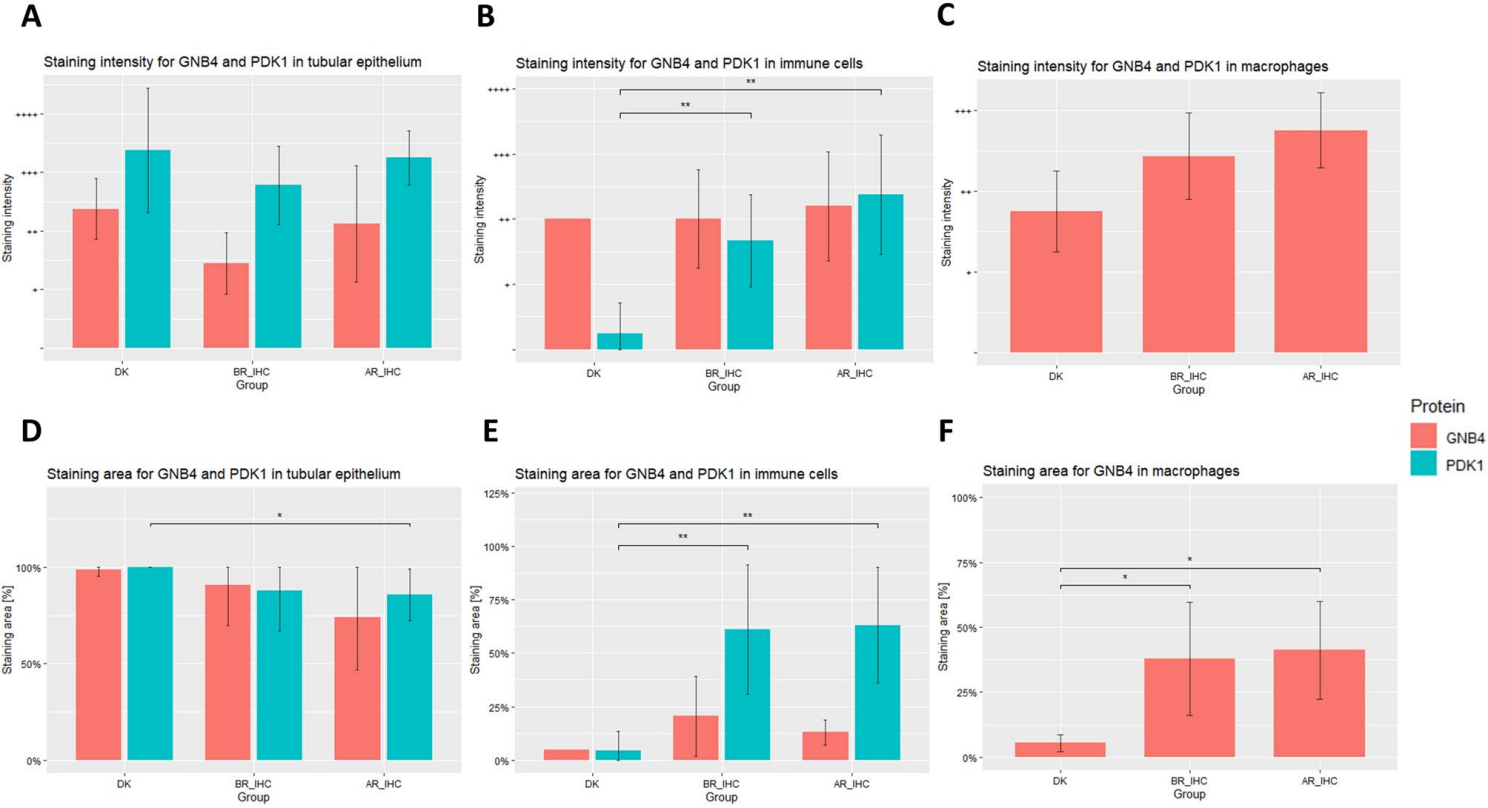
Differences in immunohistochemical staining for PDK1 and GNB4 in kidney tissue samples. **A, B, C** Staining intensity in the tubular epithelium, immune cells, and macrophages. **D, E, F** Staining area in the tubular epithelium, immune cells, and macrophages. Results for PDK1 in macrophages have been omitted since no visible staining could be observed. Statistical significance is denoted as: * *p* < 0.05, ** *p* < 0.01

We found that neither PDK1 nor GNB4 was significantly up-regulated in the tubular epithelium of patients with the transplant rejection (in the case of PDK1, lower intensity of the staining was noted in the BR_IHC group in comparison to DK vs AR_IHC) (Fig. 6A, 6). On the other hand, significant differences were noted among the compared groups in the case of the immune cells component of the specimen. For PDK1, both the area and intensity of staining of immune cells were significantly higher in the BR_IHC and AR_IHC groups compared to the DK (differences in the staining of GNB4 were not statistically significant when all immune cells were considered) (Fig. 6B, 6). However, in the case of GNB4, significantly stronger staining (both area and intensity) in the BR_IHC and AR_IHC groups was noted specifically for macrophages (PDK1 was not detected in macrophages) (Fig. 6C, 6).

## Discussion

Identification of a reliable molecular biomarker associated with the risk of T cell-mediated rejection (TCMR) remains a critical challenge in the field of kidney transplantation. Although several studies have proposed hypothetical biomarker signatures based on protein components of urine or plasma, their findings have not been confirmed in larger clinical trials [15], with analyses specific for tissue biopsies being scarce [23, 24]. In this study, we have performed proteomic profiling of kidney specimens (FFPE tissue biopsy samples) from patients with acute or borderline TCMR and without transplant rejection. Using an MS-based approach, we were able to identify a set of proteins differentiating the experimental groups. Their functional analysis revealed a strong correlation with immune system functioning, *e.g.*, the process of cytokine signaling, which is expected to be overrepresented in studies of TCMR [25–27]. Moreover, overrepresentation of the term “RHO GTPase effectors”, which control cell motility, has been previously linked with reduced kidney function and immune cell activation [28, 29]. A hypothetical multicomponent signature was proposed that showed a strong predictive value of NR vs AR or BR. This signature included CLYBL that had already been identified as a predictive factor in kidney transplant rejection [30, 31]. Other components of this hypothetical signature, FBLN5, GSTT1, TXN, and ITIH4, have been connected to kidney injury, renal cancer progression, and immune system activation [32–36]. TXN protein specifically differentiated AR and NR groups in a study by Feng et al. using kidney biopsies [23]. In that same study, AGXT2 (protein with a high degree of similarity to AGXT) was also reported as differentiating.

Kidney transplant rejection is a process tightly regulated by the immune system. T cell-mediated rejection (TCMR) is, by definition, characterized by T cell infiltration at the rejection site, a condition that is further exacerbated by macrophage activation [5, 37, 38]. It has been previously shown that monitoring of a kidney status with immunological markers using an MS approach is possible, which is exemplified by the assessment of CD45 (protein expressed on all leukocytes) [24]. However, since a typical proteomics protocol for an MS analysis applies to lysates of heterogeneous tissue specimens, the information on the cell-type-specificity of detected proteins is missed. Therefore, for the validation study, we have chosen the immunohistochemical approach (instead of a Western Blot analysis), which enables us to address the specific cellular and sub-cellular context of protein localization. With this approach, using a partly independent set of specimens, we confirmed enhanced accumulation of two candidate biomarkers in specimens representative of acute and borderline TCMR (compared to the kidney of healthy donors). PDK1 was upregulated in immune cells generally, while GNB4 was upregulated in macrophages of patients who rejected the transplant (noteworthy, differences between groups of samples were negligible when the tubular kidney epithelium was addressed). This finding adds a new context to the connections between the immune system and a kidney transplant rejection. GNB4 has been previously reported to be associated with the immune system functioning; however, the role of either GNB4 or PDK1 in processes related to kidney transplantation is uncommon [39]. Although upregulation of CD73 or AGXT in kidney specimens of patients who rejected the transplant was not confirmed using immunohistochemistry, it should be noted that CD73 is a known immune checkpoint molecule playing a costimulatory role for T lymphocytes [40–43].

## Conclusions

We have identified a multi-component panel of proteins putatively associated with kidney transplant rejection. Moreover, using immunohistochemistry analysis, we validated two biomarker candidates, PDK1 and GNB4, that accumulated in the kidney of patients with pathologic diagnosis of both acute and borderline TCMR, which are associated with the functions of immune cells and macrophages. Hence, a new approach to the monitoring of transplanted kidney status could be proposed based on examination of a biopsy. However, further investigation is required to verify the clinical applicability of findings, particularly in the context of identifying borderline changes requiring additional clinical intervention.

## Study limitations

All kidney allograft samples used in this retrospective study were remnant clinical samples. The biopsies were performed over a period of nine years, during which several major revisions of the Banff Classification of Kidney Allograft Pathology were made, including the criteria defining borderline changes, which could have affected the final diagnosis [7]. The set of samples used in the MS-based proteomic analysis and immunohistochemistry staining only partially overlapped. This was due to the low availability of the studied material, where the volume of kidney biopsies is minimized for patient safety. On the other hand, performing the IHC analysis for independent samples enhanced the strength of the validation study. The localization of the analyzed proteins in tubular epithelium, immune cells, and/or macrophages was analyzed by IHC without co-staining of specific markers of these structures (based on morphological features determined in situ by brightfield microscopy).

Taking the above-mentioned limitations into account, follow-up studies should be performed on a set of samples obtained in a relatively short period of time (i.e., less than 9 years), where Banff classification criteria remained constant. Moreover, validation studies using IHC should include immune cell co-staining.

## Supplementary Information

Supplementary Material 1

Supplementary Material 2

Supplementary Material 3

Supplementary Material 4

Supplementary Material 5

Supplementary Material 6

## Data Availability

The mass spectrometry datasets generated and analyzed during the current study are available on ProteomeXchange via the PRIDE partner repository [44, 45] with the dataset identifier PXD064523. Immunohistochemistry results analyzed during the current study are available from the corresponding author upon reasonable request.

## Abbreviations

AGXT: Alanine–glyoxylate aminotransferase
AmBIC: Ammonium bicarbonate
AR: Acute rejection
AUC: Area under curve
BR: Borderline rejection
CD73: 5′-Nucleotidase
DK: Donor kidney
FDR: False-discovery rate
FFPE: Formalin-fixed paraffin-embedded [tissue]
GNB4: Guanine nucleotide-binding protein subunit beta-4
H&E: Hematoxylin and eosin [staining]
HCD: Higher energy collisional dissociation
IHC: Immunohistochemistry
LC–MS/MS: Liquid chromatography–tandem mass spectrometry
MS: Mass spectrometry
NR: No rejection
PDK1: PDH kinase 1
ROC: Receiver operating characteristic [curve]
TCMR: T-cell mediated rejection

## References

[1] Starzl TE. History of clinical transplantation. World J Surg. 2000;24(7):759–82. 10833242 10.1007/s002680010124 PMC3091383

[2] Cooper JE. Evaluation and treatment of acute rejection in kidney allografts. Clin J Am Soc Nephrol CJASN. 2020;15(3):430–8. 32066593 10.2215/CJN.11991019 PMC7057293

[3] Lusco MA, Fogo AB, Najafian B, Alpers CE. AJKD atlas of renal pathology: acute T-cell–mediated rejection. Am J Kidney Dis. 2016;67(5):e29-30. 27091022 10.1053/j.ajkd.2016.03.004

[4] Naesens M, Roufosse C, Haas M, Lefaucheur C, Mannon RB, Adam BA, et al. The Banff 2022 kidney meeting report: reappraisal of microvascular inflammation and the role of biopsy-based transcript diagnostics. Am J Transplant. 2024;24(3):338–49. 38032300 10.1016/j.ajt.2023.10.016

[5] Loupy A, Haas M, Roufosse C, Naesens M, Adam B, Afrouzian M, et al. The Banff 2019 kidney meeting report (I): updates on and clarification of criteria for T cell– and antibody-mediated rejection. Am J Transplant. 2020;20(9):2318–31. 32463180 10.1111/ajt.15898 PMC7496245

[6] Nankivell BJ, Agrawal N, Sharma A, Taverniti A, P’Ng CH, Shingde M, et al. The clinical and pathological significance of borderline T cell–mediated rejection. Am J Transplant. 2019;19(5):1452–63. 30501008 10.1111/ajt.15197

[7] Loupy A, Mengel M, Haas M. Thirty years of the International banff classification for allograft pathology: the past, present, and future of kidney transplant diagnostics. Kidney Int. 2022;101(4):678–91. 34922989 10.1016/j.kint.2021.11.028

[8] de Freitas DG, Sellarés J, Mengel M, Chang J, Hidalgo LG, Famulski KS, et al. The nature of biopsies with “borderline rejection” and prospects for eliminating this category. Am J Transplant. 2012;12(1):191–201. 21992503 10.1111/j.1600-6143.2011.03784.x

[9] Mehta RB, Tandukar S, Jorgensen D, Randhawa P, Sood P, Puttarajappa C, et al. Early subclinical tubulitis and interstitial inflammation in kidney transplantation have adverse clinical implications. Kidney Int. 2020;98(2):436–47. 32624181 10.1016/j.kint.2020.03.028

[10] Heilman RL, Nijim S, Chakkera HA, Devarapalli Y, Moss AA, Mulligan DC, et al. Impact of acute rejection on kidney allograft outcomes in recipients on rapid steroid withdrawal. J Transplant. 2011;2011(1):583981. 21647349 10.1155/2011/583981 PMC3103882

[11] Masin-Spasovska J, Spasovski G, Dzikova S, Petrusevska G, Dimova B, Lekovski L, et al. The evolution of untreated borderline and subclinical rejections at first month kidney allograft biopsy in comparison with histological changes at 6 months protocol biopsies. Prilozi. 2005;26(1):25–33. 16118612

[12] Thierry A, Thervet E, Vuiblet V, Goujon JM, Machet MC, Noel LH, et al. Long-term impact of subclinical inflammation diagnosed by protocol biopsy one year after renal transplantation. Am J Transplant. 2011;11(10):2153–61. 21883902 10.1111/j.1600-6143.2011.03695.x

[13] Wang C, Feng G, Zhao J, Xu Y, Li Y, Wang L, et al. Screening of novel biomarkers for acute kidney transplant rejection using DIA-MS based proteomics. PROTEOMICS – Clinical Appl. 2024;18(3):2300047. 10.1002/prca.202300047 38215274

[14] Heidari SS, Nafar M, Kalantari S, Tavilani H, Karimi J, Foster L, et al. Urinary epidermal growth factor is a novel biomarker for early diagnosis of antibody mediated kidney allograft rejection: A urinary proteomics analysis. J Proteomics. 2021;30(240):104208. 10.1016/j.jprot.2021.104208 33785428

[15] Gwinner W, Karch A, Braesen JH, Khalifa AA, Metzger J, Naesens M, et al. Noninvasive diagnosis of acute rejection in renal transplant patients using mass spectrometric analysis of urine samples: a multicenter diagnostic phase III trial. Transplant Direct. 2022;8(5):e1316. 35434282 10.1097/TXD.0000000000001316 PMC9005257

[16] Geoui T, Urlaub H, Plessmann U, Porschewski P. Extraction of proteins from formalin-fixed, paraffin-embedded tissue using the qproteome extraction technique and preparation of tryptic peptides for liquid chromatography/mass spectrometry analysis. Current Protocls Mol Biol. 2010;90(1):10–27. 10.1002/0471142727.mb1027s90 20373500

[17] Wiśniewski JR, Gaugaz FZ. Fast and sensitive total protein and Peptide assays for proteomic analysis. Anal Chem. 2015;87(8):4110–6. 25837572 10.1021/ac504689z

[18] Conover WJ. Practical nonparametric statistics. 3rd ed. New York: Wiley; 1998.

[19] Jonckheere AR. A distribution-free k-sample test against ordered alternatives. Biometrika. 1954;41(1–2):133–45.

[20] Pallant J. SPSS Survival Manual: A Step by Step Guide to Data Analysis Using SPSS for Windows. 4th ed. McGraw Hill Open University Press; 2010.

[21] Cohen J. Statistical Power Analysis for the Behavioral Sciences. 2nd ed. Hillsdale; 1988.

[22] Armitage P. Tests for Linear Trends in Proportions and Frequencies. Biometrics. 1955;11(3):375.

[23] Fang F, Liu P, Song L, Wagner P, Bartlett D, Ma L, et al. Diagnosis of T-cell-mediated kidney rejection by biopsy-based proteomic biomarkers and machine learning. Front Immunol. 2023;6(14):1090373. 10.3389/fimmu.2023.1090373 PMC9939643 36814924

[24] Hofstraat R, Marx K, Blatnik R, Claessen N, Chojnacka A, Peters-Sengers H, et al. Highly Repeatable Tissue Proteomics for Kidney Transplant Pathology: Technical and Biological Validation of Protein Analysis using LC-MS/MS. bioRxiv; 2024. p 2024.06.14.599091. 10.1016/j.labinv.2026.106073 41548757

[25] Schmouder RL, Kunkel SL. The cytokine response in renal allograft rejection. Nephrol Dial Transplant. 1995;10(supp1):36–43. 7617280 10.1093/ndt/10.supp1.36

[26] De Serres SA, Mfarrej BG, Grafals M, Riella LV, Magee CN, Yeung MY, et al. Derivation and validation of a cytokine-based assay to screen for acute rejection in renal transplant recipients. Clin J Am Soc Nephrol. 2012;7(6):1018. 22498498 10.2215/CJN.11051011 PMC3362312

[27] Karczewski J, Karczewski M, Glyda M, Wiktorowicz K. Role of TH1/TH2 cytokines in kidney allograft rejection. Transplant Proc. 2008;40(10):3390–2. 19100396 10.1016/j.transproceed.2008.07.125

[28] Biro M, Munoz MA, Weninger W. Targeting Rho-GTPases in immune cell migration and inflammation. Br J Pharmacol. 2014;171(24):5491–506. 24571448 10.1111/bph.12658 PMC4282076

[29] Matsuda J, Asano-Matsuda K, Kitzler TM, Takano T. Rho GTPase regulatory proteins in podocytes. Kidney Int. 2021;99(2):336–45. 33122025 10.1016/j.kint.2020.08.035

[30] Kurian SM, Velazquez E, Thompson R, Whisenant T, Rose S, Riley N, et al. Orthogonal comparison of molecular signatures of kidney transplants with subclinical and clinical acute rejection: equivalent performance is agnostic to both technology and platform. Am J Transplant. 2017;17(8):2103–16. 28188669 10.1111/ajt.14224 PMC5519433

[31] Kurian SM, Williams AN, Gelbart T, Campbell D, Mondala TS, Head SR, et al. Molecular classifiers for acute kidney transplant rejection in peripheral blood by whole genome gene expression profiling. Am J Transplant. 2014;14(5):1164–72. 24725967 10.1111/ajt.12671 PMC4439107

[32] Zhang M, Chen F, Feng S, Liu X, Wang Z, Shen N, et al. FBLN5 as one presumably prognostic gene potentially modulating tumor immune microenvironment for renal clear cell carcinoma in children and young adults. Pharmacogenomics Pers Med. 2024;19(17):27–40. 10.2147/PGPM.S442803 PMC10804877 38264064

[33] Huang W, Shi H, Hou Q, Mo Z, Xie X. GSTM1 and GSTT1 polymorphisms contribute to renal cell carcinoma risk: evidence from an updated meta-analysis. Sci Rep. 2015;5(1):17971. 26656529 10.1038/srep17971 PMC4677290

[34] Li P, Li D, Lu Y, Pan S, Cheng F, Li S, et al. GSTT1/GSTM1 deficiency aggravated cisplatin-induced acute kidney injury via ROS-triggered ferroptosis. Front Immunol. 2024;25(15):1457230. 10.3389/fimmu.2024.1457230 PMC11461197 39386217

[35] Beothe T, Docs J, Kovacs G, Peterfi L. Increased level of TXNIP and nuclear translocation of TXN is associated with end stage renal disease and development of multiplex renal tumours. BMC Nephrol. 2024;17(25):227. 10.1186/s12882-024-03653-4 PMC11256699 39020292

[36] Luczak M, Formanowicz D, Marczak Ł, Pawliczak E, Wanic-Kossowska M, Figlerowicz M, et al. Deeper insight into chronic kidney disease-related atherosclerosis: comparative proteomic studies of blood plasma using 2DE and mass spectrometry. J Transl Med. 2015;13(1):20. 25622820 10.1186/s12967-014-0378-8 PMC4316657

[37] Abdullah EL, Jalalonmuhali M, Ng KP, Jamaluddin FA, Lim SK. The role of lymphocyte subset in predicting allograft rejections in kidney transplant recipients. Transplant Proceed. 2022;54(2):312–9. 10.1016/j.transproceed.2022.01.009 35246329

[38] Halloran PF. T cell-mediated rejection of kidney transplants: a personal viewpoint. Am J Transplant. 2010;10(5):1126–34. 20346061 10.1111/j.1600-6143.2010.03053.x

[39] Liu B, Chen L, Huang H, Huang H, Jin H, Fu C. Prognostic and immunological value of GNB4 in gastric cancer by analyzing TCGA database. Dis Markers. 2022;16(2022):1–16. 10.1155/2022/7803642 PMC9225895 35756485

[40] Zhan J, Huang L, Niu L, Lu W, Sun C, Liu S, et al. Regulation of CD73 on NAD metabolism: unravelling the interplay between tumour immunity and tumour metabolism. Cell Commun Signal. 2024;22(1):387. 39090604 10.1186/s12964-024-01755-y PMC11292923

[41] Chen S, Wainwright DA, Wu JD, Wan Y, Matei DE, Zhang Y, et al. Cd73: an emerging checkpoint for cancer immunotherapy. Immunotherapy. 2019;11(11):983–97. 31223045 10.2217/imt-2018-0200 PMC6609898

[42] Antonioli L, Pacher P, Vizi ES, Haskó G. CD39 and CD73 in immunity and inflammation. Trends Mol Med. 2013;19(6):355–67. 23601906 10.1016/j.molmed.2013.03.005 PMC3674206

[43] Schneider E, Winzer R, Rissiek A, Ricklefs I, Meyer-Schwesinger C, Ricklefs FL, et al. CD73-mediated adenosine production by CD8 T cell-derived extracellular vesicles constitutes an intrinsic mechanism of immune suppression. Nat Commun. 2021;12(1):5911. 34625545 10.1038/s41467-021-26134-w PMC8501027

[44] Deutsch EW, Bandeira N, Perez-Riverol Y, Sharma V, Carver JJ, Mendoza L, et al. The proteomexchange consortium at 10 years: 2023 update. Nucleic Acids Res. 2023;51(D1):D1539–48. 36370099 10.1093/nar/gkac1040 PMC9825490

[45] Perez-Riverol Y, Bandla C, Kundu DJ, Kamatchinathan S, Bai J, Hewapathirana S, et al. The PRIDE database at 20 years: 2025 update. Nucleic Acids Res. 2025;53(D1):D543–53. 39494541 10.1093/nar/gkae1011 PMC11701690

