# Supplementary figures and images for: Biopsy proteome-based classification of T cell-mediated kidney allograft rejection
Source: J Transl Med. 2025 Oct 21;23:1139. doi: 10.1186/s12967-025-07116-8 (PMC12539011; doi:10.1186/s12967-025-07116-8)

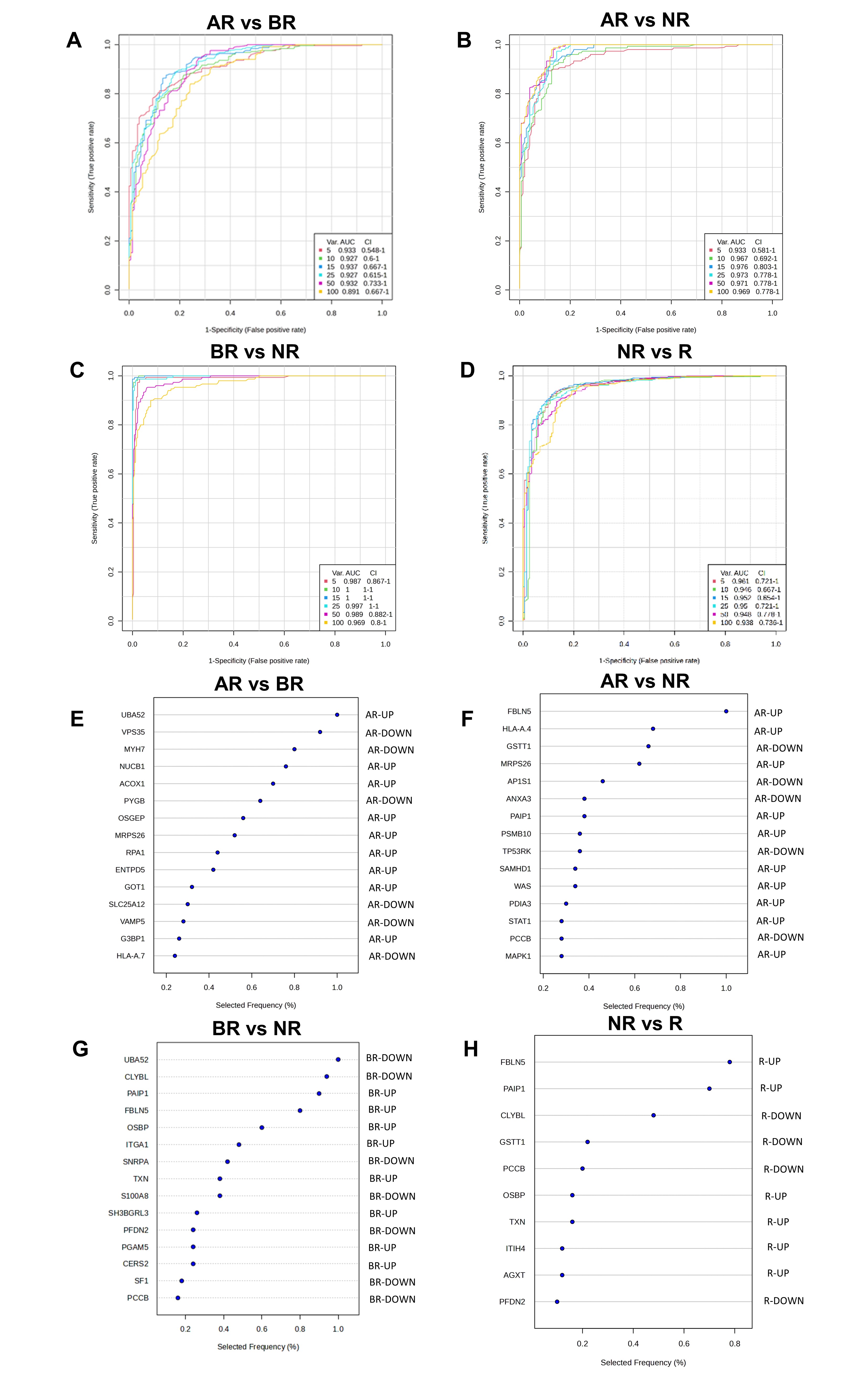

Supplement: Supplementary file 4 — Supplementary Material 4 [file 12967_2025_7116_MOESM4_ESM.jpeg]

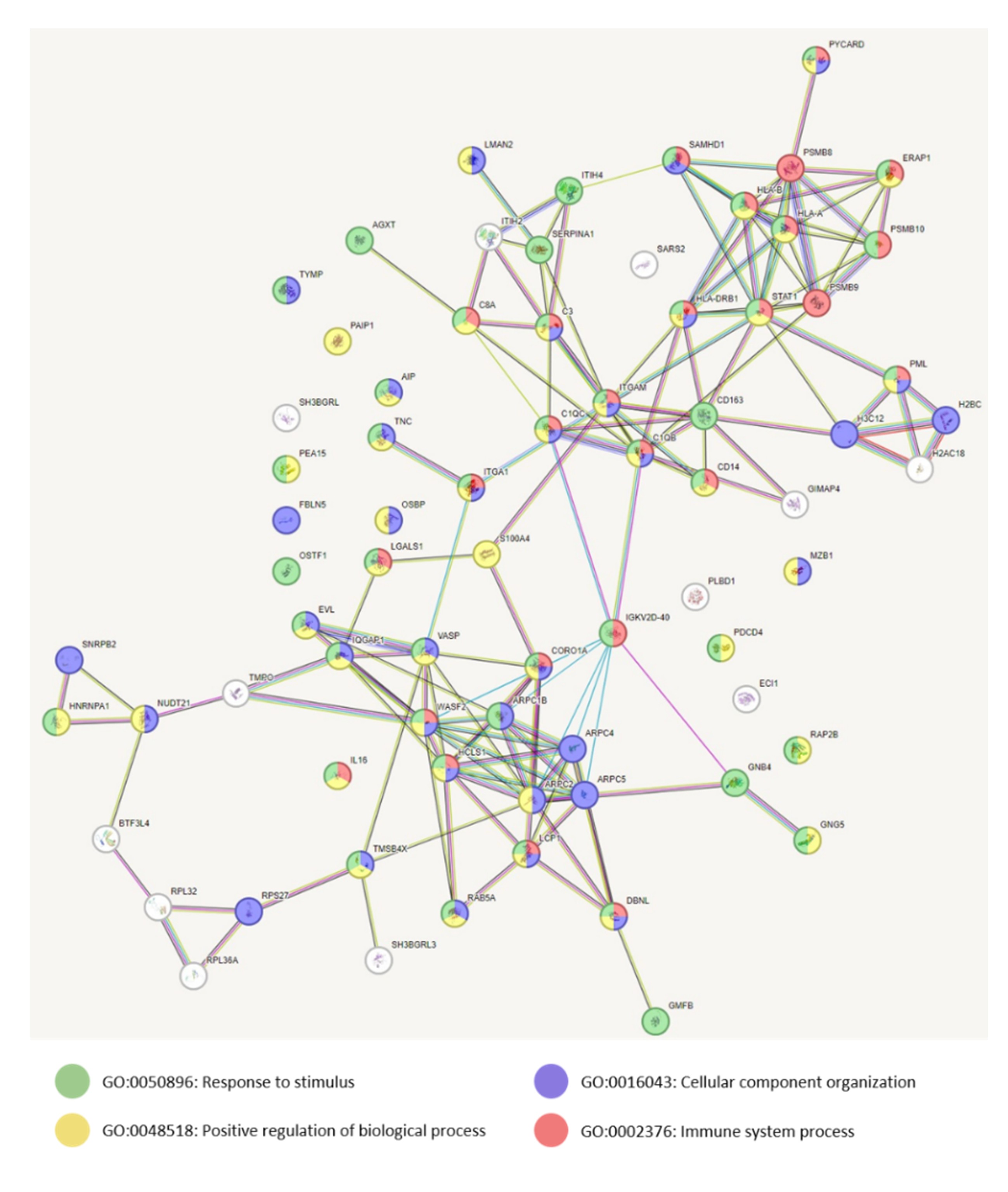

Supplement: Supplementary file 6 — Supplementary Material 6 [file 12967_2025_7116_MOESM6_ESM.jpeg]
